# Diotic and Dichotic Mechanisms of Discrimination Threshold in Musicians and Non-Musicians

**DOI:** 10.3390/brainsci11121592

**Published:** 2021-11-30

**Authors:** Devin Inabinet, Jan De La Cruz, Justin Cha, Kevin Ng, Gabriella Musacchia

**Affiliations:** 1 Department of Audiology, University of the Pacific, San Francisco, CA 94103, USA; d_inabinet@u.pacific.edu (D.I.); jdelacruz@pacific.edu (J.D.L.C.); j_cha@u.pacific.edu (J.C.); k_ng@u.pacific.edu (K.N.); 2 Otolaryngology Head and Neck Surgery, Stanford University, Palo Alto, CA 94303, USA

**Keywords:** musician, pitch, discrimination, dichotic, music

## Abstract

The perception of harmonic complexes provides important information for musical and vocal communication. Numerous studies have shown that musical training and expertise are associated with better processing of harmonic complexes, however, it is unclear whether the perceptual improvement associated with musical training is universal to different pitch models. The current study addresses this issue by measuring discrimination thresholds of musicians (*n* = 20) and non-musicians (*n* = 18) to diotic (same sound to both ears) and dichotic (different sounds to each ear) sounds of four stimulus types: (1) pure sinusoidal tones, PT; (2) four-harmonic complex tones, CT; (3) iterated rippled noise, IRN; and (4) interaurally correlated broadband noise, called the “Huggins” or “dichotic” pitch, DP. Frequency difference limens (DLF) for each stimulus type were obtained via a three-alternative-forced-choice adaptive task requiring selection of the interval with the highest pitch, yielding the smallest perceptible fundamental frequency (F0) distance (in Hz) between two sounds. Music skill was measured by an online test of musical pitch, melody and timing maintained by the International Laboratory for Brain Music and Sound Research. Musicianship, length of music experience and self-evaluation of musical skill were assessed by questionnaire. Results showed musicians had smaller DLFs in all four conditions with the largest group difference in the dichotic condition. DLF thresholds were related to both subjective and objective musical ability. In addition, subjective self-report of musical ability was shown to be a significant variable in group classification. Taken together, the results suggest that music-related plasticity benefits multiple mechanisms of pitch encoding and that self-evaluation of musicality can be reliably associated with objective measures of perception.

## 1. Introduction

Musical training is associated with better frequency encoding and harmonic complex perception (for review see [1]). The perception of frequencies and harmonics is often referred to as pitch perception and in music, is the quality of sound that most strongly defines the melodic contour. Each note in a melody has a pitch that is related to the frequencies and harmonics of an instrumental sound. The underlying mechanisms are still under debate, predominantly due to the variety of acoustic components that can give rise to the sensation of pitch (for review see [2]). Despite the controversy over underlying mechanisms, a person’s pitch perception of sinusoidal and harmonic sounds can be reliably measured by the difference limen for frequency (DLF), or the smallest perceptible change from a center frequency [3,4]. In general, normal-hearing listeners can perceive a change in as little as 2–3% from a center frequency under optimal listening conditions and using sounds with high pitch salience [5]. Musicians can detect even smaller pitch changes, sometimes so minute that the change is undetectable by otherwise normal-hearing non-musicians [2,6,7]. Not surprisingly, increased acuity in musicians is not limited to musical sounds but extends to perception and processing of speech [8], non-speech ([6] for review [9]) and non-native language sounds [10].

A prevalent hypothesis is that musical training improves underlying auditory processing mechanisms that give rise to pitch perception. However, the auditory system utilizes several mechanisms to encode pitch-related acoustics and it is unclear which ones are most improved with music training. One way the auditory system represents sound is by via a “temporal code” in which alteration of neuronal spike timing conveys information about stimulus attributes. One of the most well-known time codes is phase-locking; in which a population of neurons work together to form a temporal pattern that matches the period, or frequency inverse, of a sound (for review see [11]). The phase-locked temporal pattern can serve as the basis for higher order computations that gives rise to pitch perception [12,13]. Music practice and performance could activate and strengthen the temporal synchrony of these networks, thereby improving representation and higher-order computation acuity. Another mechanism to encode sound, called “place code”, functions such that different frequencies activate discrete regions of the inner ear and subsequent nuclei, producing a tonotopic map of frequencies at each processing station (for review, see [14]). For example, the perception of pitch rises as the region of maximal activation on the basilar membrane moves closer to the base of the cochlea. Music training could generate more precise and definite tonotopic maps due to top-down modulation induced by the increased prevalence and relevance of sounds in the environment, perhaps similar to animal models [15,16]. Finally, a pitch perception can be generated by presenting different sound components to each ear, creating a dichotic (binaural) or combined estimation of the sound’s pitch [17,18]. Music training could alter the anisotropy between the left and right ears either through repeated computation of binaural timing differences during localization of instruments in their group [19], or by focusing on different portions of the spectrum in music listening [20].

The auditory system may use any encoding strategy presented above to encode sound. However, the magnitude of each strategy’s contribution to perception depends on what features are present in the stimulus. For example, spectrally simple sounds less than 2 kHz elicit widely spaced, narrow bands of activity on the cochlear partition that are easily represented in tonotopic map and are likely be dominated by the place code strategy of encoding. On the other hand, sounds that elicit a pitch perception despite the lack of a well-defined places of maximal vibration are likely to rely more on temporal encoding strategies.

The working hypothesis that motivated this study was that music training promotes plasticity in specific pitch-related encoding mechanisms. Particularly, we posited that sounds that were more reliant on temporal encoding would be impacted the most because playing music requires considerable focus on sound timing. To test this, we measured DLFs in musicians and non-musicians using four different types of sounds with different pitch-related acoustics.

The results of this study should inform us as to whether the plasticity associated with musical training is specific to different degrees of temporal, place, dichotic and diotic encoding. Creation of the sounds to conduct the experiment was inspired by work describing how different degrees of stimulus manipulations can be elicited by pitch percepts [21]. In order to assess musical skill, all participants took an online musical test for pitch, melody and timing measurements and filled out a questionnaire that probed duration of musical training and subjective self-reports of musical skill and listening habits.

## 2. Materials and Methods

PARTICIPANTS: 38 individuals with audiometric thresholds within normal limits (<25 dB HL for 0.25, 0.5, 1, 2, 3, 4, 6 and 8 kHz, assessed at time of testing) and no history of neurological disorders participated in the study. A total of 20 of our participants self-identified as female, 18 self-identified as male. Previous research has shown that music-related brain plasticity is most effective when people begin playing music early, continue, and are currently practicing [22,23,24]. Therefore, subject inclusion criteria in the musician (MU) group included: (1) self-identification as a musician via questionnaire and reported current involvement in musical activities, (2) self-report of music training initiation before high school (e.g., before Grade 9, ages 14–15) and (3) a total of at least 5 years in formal music education. A total of 20 subjects fulfilled the criteria for MU group inclusion, with the remainder 18 subjects grouped into non-musicians (NM). Regarding language experience, 37 participants were native English speakers and English was not the native language for one NM. Eight out 18 NMs and 11 out of 20 MUs spoke more than one language fluently. Further, 15 out of 18 NMs and 16 out of 20 MUs were students. Group characteristics of age, music education, self-ratings and objective measures of musical skill (i.e., online aptitude test, for description see below) are presented in Table 1.

QUESTIONNAIRE: Participants’ musical history was collected through a questionnaire probing a range of information regarding subjective aptitude and measures of musicianship after experimental testing. We used the following details and scale-based ratings to correlate with objective performance on psychoacoustic measures: (1) Musician self-identification (e.g., “Are you a musician?”), (2) self-report of music listening frequency on a scale of 1–9, (3) self-report of musical skill on a scale of 1–9, (4) age of music start, and (5) years of consistent practice. Group means and standard deviations are shown in Table 1.

MUSICAL APTITUDE TEST: Individuals completed an online test prior to experimental testing through the International Laboratory for Brain, Music, and Sound Research (BRAMS/) that allows for the assessment of the functioning of each musical component: (1) melody, (2) timing, and (3) pitch ability. The online test battery is based on the Montreal battery for evaluation of amusia (MBEA) and consists of musical phrases that vary along the melody, timing, or pitch dimension [25]. During the MBEA, listeners perform a task to determine whether two presented musical phrases are the same or different. In the melody test, for example, the two choices may consist of an original melodic contour and a scale- or contour-violated alternate. The output of the online test is a percentage correct for each task, in addition to the average of all three categories. These percentages were recorded and utilized in this study. Group means and standard deviations are shown in Table 1.

**Figure 1:**
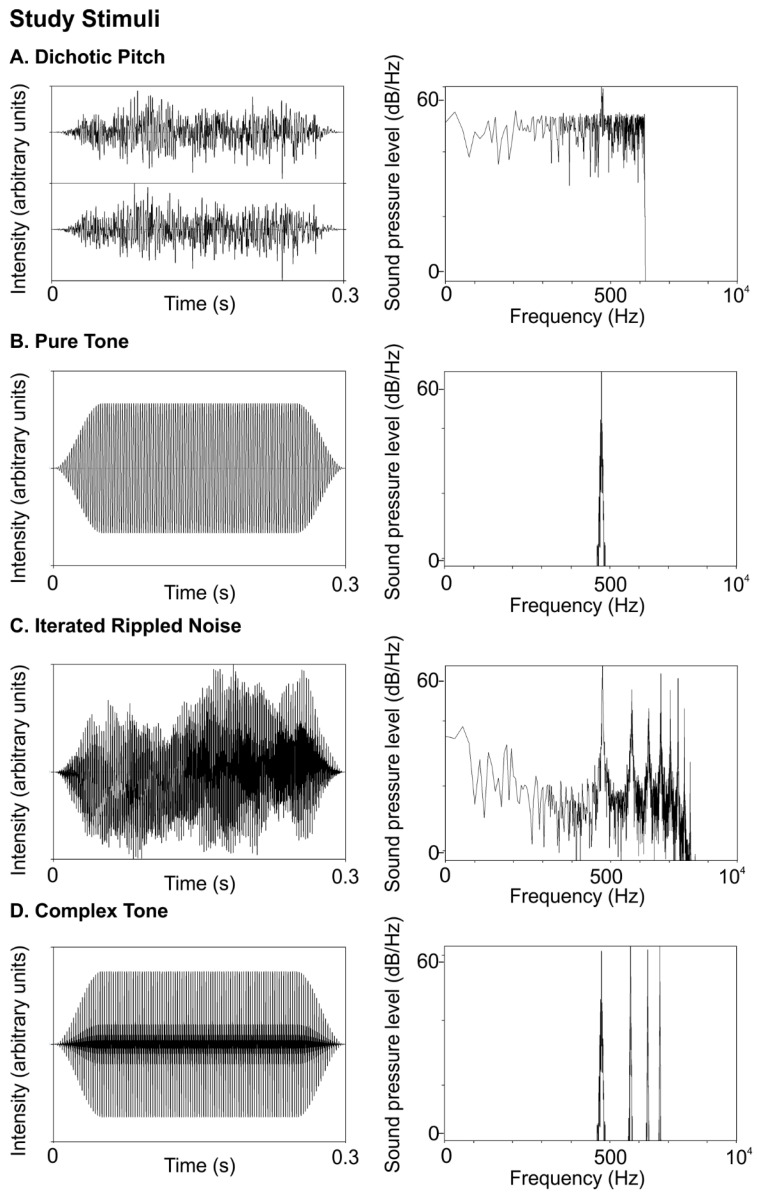
Study stimuli. Each row shows the 440 Hz stimulus waveform (left panel) and spectrum on a logarithmic frequency scale (right panel). (**A**). Dichotic pitch with an interaural phase shift of 440 Hz, (**B**). Pure tone, (**C**). Iterated rippled noise with a 64 iteration of delay and add at 1/440 s. (**D**). Complex tone with three overtone harmonics.

STIMULI: Sounds were 300 ms in duration, with two 60-ms raised cosine ramps for onset and offset. Figure 1 shows time waveforms (left panels) and frequency spectra (right panels) for the 440 Hz (standard) stimuli used in the study. Further, 440 Hz was chosen because it is a familiar musical note (A4) that elicits strong phase-locking.

In order to test binaural mechanisms, we created a dichotic pitch (DP) stimulus, often called “Huggins’ pitch,” which consists of dissimilar right and left inputs to make a dichotic estimation of a sound’s pitch [17,18]. DP stimuli were created with the Binaural Auditory Processing Toolbox for MATLAB8 using a transition width of 16%. DP sounds were made of white noise, diotic at all frequencies except for a narrow band at the F0 (440 Hz), over which the interaural phase transitioned progressively through 360°. Individuals were familiarized with DP perception through five online demonstrations by Robert Dougherty https://web.stanford.edu/~bobd/cgi-bin/research/dpDemos/ (accessed on 13 September 2018), [26].

In contrast, a pure tone (PT), shown in Panel B of Figure 1 is the product of a sinusoidal function. Sinusoids are likely to be encoded via place code mechanisms because they elicit narrow bands of maximal activation at specific places in the tonotopic map of the cochlea [27]. At lower frequencies (<~2 kHz) elicit additional phase-locked temporal codes at the frequency’s period. Pure tones consisted of sinusoids at a fundamental frequency (F0) of 440 Hz.

We also tested an iterated noise (IRN) stimulus which evokes a pitch perception that is primarily reliant on temporal information [28,29,30]. IRN stimuli, shown in Figure 1C, were created from Gaussian broadband noise filtered from 80–3000 Hz with 64 iterations of delay and add durations at the inverse of the F0 (440 Hz) (code adapted with permission from [30]). The temporal regularity imposed on broadband noise gives rise to the perception of pitch despite low spectral content.

Finally, we used a complex tone with three harmonic overtones (CT), which, in comparison to our other stimuli, most closely resembles the sound a musical instrument makes and relies on a combination of place and temporal codes. Complex tones (Figure 1D) consisted of a four-harmonic complex (h1–h4) with equal amplitude and the same F0 (440 Hz).

EXPERIMENTAL TASK AND PROCEDURE: Listeners were seated comfortably in front of a Surface Pro laptop computer in a soundproof room. Stimuli were presented through Sennheiser headphones using the Psychoacoustics MATLAB toolbox (Soranzo and Grassi, 2014) in a three-alternative forced choice paradigm in which subjects were asked to press the keyboard to tell which of three successive intervals contained the higher sounding pitch. In each trial, two out of three of the sounds were the standard frequency, 440 Hz, and one was always higher than the standard by a factor of Δ*f*.

To estimate the DLF threshold, we employed a transformed adaptive staircase procedure (Levitt, 1971), following a two-down/one-up algorithm (TwoDownOneUp in the PSYCHOACOUSTICS Graphical User Interface). This method uses the previous one or two responses to select the next trial frequency and tracks threshold at 70.7% correct. Specifically, two correct responses in a row decreased the frequency by a factor, Δ*f*, making the task more difficult and one incorrect response increased the frequency by Δ*f*, making the task easier. Two values of Δ*f*, were used within a single run: Δ*f* = 2 to approach threshold quickly and Δ*f* = sqrt(2) to remain near threshold. The initial value of Δ*f* was 100 Hz.

DLF threshold for each run was calculated by averaging the last four out of 12 reversals. A reversal pattern is one in which the subject changes their response. For example, as long as a subject can correctly identify the interval with the highest pitch, Δ*f* will be reduced and no reversal in response pattern occurs. At some point the change in Δ*f* will be below the subject’s sensory threshold, and they will guess incorrectly. At that point the standard frequency will be increased by Δ*f*. This constitutes a reversal pattern because the subject has gone from a correct response to an incorrect one. Similarly, a reversal occurs when a subject changes their response pattern from incorrect to correct.

CT, PT, HP and IRN conditions were presented in separate blocks, with each block consisting of four runs with block order was pseudorandomized across subjects. Mean DLF for each condition was calculated by averaging thresholds across the four runs in each block. Standard deviation across the four runs in each block was also calculated to estimate threshold variability in each condition. The standard deviation across runs gives an estimate of within-session change in threshold, or a broad measure of how consistent each group performs. The number of trials to threshold in each run were used to calculate a mean trials value for each condition. This measure gives an estimate of how fast each group converged upon threshold. Mean DLF, standard deviation and mean trials were subsequently used in data analysis.

DATA ANALYSIS: Tests of normality were computed on all variables. Results of these tests showed that the pairs of MU and NM distributions were not significantly different from normal according to Shapiro–Wilk tests (*p* < 0.01), except for SR Musical Skill and BRAMS Pitch score (see Supplementary Table S1). Examination of the detrended SR musical skill scores showed that one NM rated themselves > 1 standard deviation from normal and one MU rated themselves > −1 standard deviation from normal. Examination of the detrended BRAMS pitch scores showed that one individual from each group scored > −1 standard deviation from normal. Given that a skew in distribution was observed for two measures, we provide observed power for each test and only conducted tests that were robust to the assumption of normality [31,32].

Statistical analyses were conducted using SPSS. Between- and within-group comparisons were assessed using mixed-model repeated-measures ANOVAs with the subsequent post hoc tests when appropriate.

To examine the question of a relationship between DLFs, musicality and self-assessment, Pearson’s r correlations were computed. Pearson’s r-values and *p*-values of the significance test are reported. In order to discover the degree to which our dependent variables discriminate between NM and MU, a discriminant function analysis with predictive classification of cases was conducted. The discriminant analysis included all four DLF thresholds, BRAMS total score and self-reported measures for a total of seven continuous, numeric variables and one categorical variable with two levels (NM, MU).

## 3. Results

### 3.1. Pitch Discrimination Thresholds

A mixed-model repeated measures ANOVA, with group (musician, non-musician) as the independent variable and stimulus type (CT, PT, IRN and DP) as the dependent variable was performed to determine the effect of group on discrimination thresholds. Results showed main effects of stimulus type; F(3108) = 38.137, *p* < 0.001, η^2^ = 0.514 and group F(1,36) = 29.205, *p* < 0.001, η ^2^ = 0.448 as well as an interaction effect; F(3108) = 11.754, *p* < 0.001, η ^2^ = 0.287. Post-hoc t-tests showed that DLF thresholds were significantly different between musicians and non-musicians for all stimulus types (*p* < 0.016).

**Figure 2:**
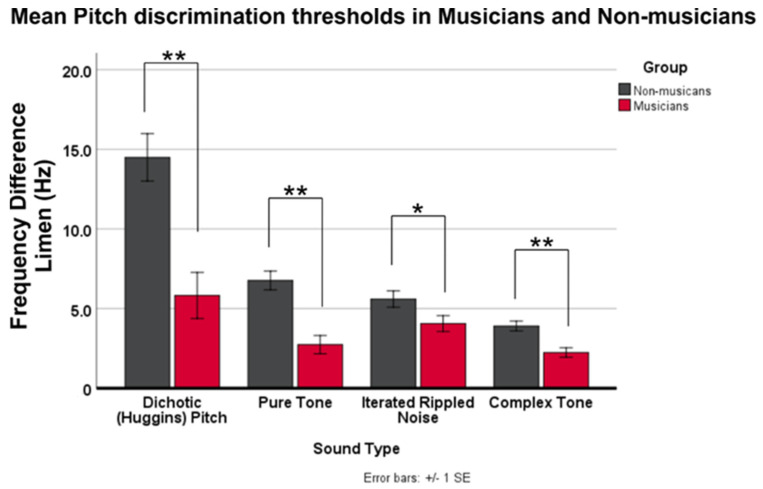
Bar graph shows mean DLF thresholds (±1 SE). Musicians have smaller (better) pitch discrimination thresholds in all conditions, relative to non-musicians (* *p* < 0.05; ** *p* < 0.01).

Group means show that musicians had lower thresholds for each stimulus type (Figure S2, Supplementary Table S2). Bar graphs in Figure 2 illustrates smaller discrimination thresholds in the musician group for all stimulus types, relative to non-musicians. Taken together, the data show that musicians can hear smaller pitch differences that non-musicians in all four pitch-evoking sound type categories, with the greatest mean difference in the DP condition and the smallest difference in the CT condition.

While threshold is a crucial index of auditory perception, reliability of threshold is also useful and can provide meaningful insight to group dynamics. Therefore, a second, more exploratory, comparison was conducted to determine intrasubject threshold variability in musicians and non-musicians. To conduct this, a mixed-model repeated measures ANOVA, with group (musician, non-musician) as the independent variable and stimulus type as the dependent variable (CT, PT, IRN and DP) was performed using the standard deviation across the four runs in each stimulus type. Results showed a main effect of stimulus type; F(3108) = 18.265, *p* < 0.001, η ^2^ = 0.337, and group F(1,36) = 11.702, *p* = 0.002, η ^2^ = 0.245 as well as an interaction between stimulus type and group; F(1,36) = 7.170, *p* = 0.011, η^2^ = 0.166. Post-hoc t-tests showed that intrasubject threshold variability was significantly different between musicians and non-musicians for DP and PT (*p* < 0.007) stimulus types, but not IRN and CT (*p* > 0.058). Examination of group means showed that threshold is less variable in MU than NM in the DP and PT condition (Supplementary Table S2).

### 3.2. Relationships between Pitch Discrimination Thresholds, Self-Reports and Musical Aptitude Measures

**Figure 3:**
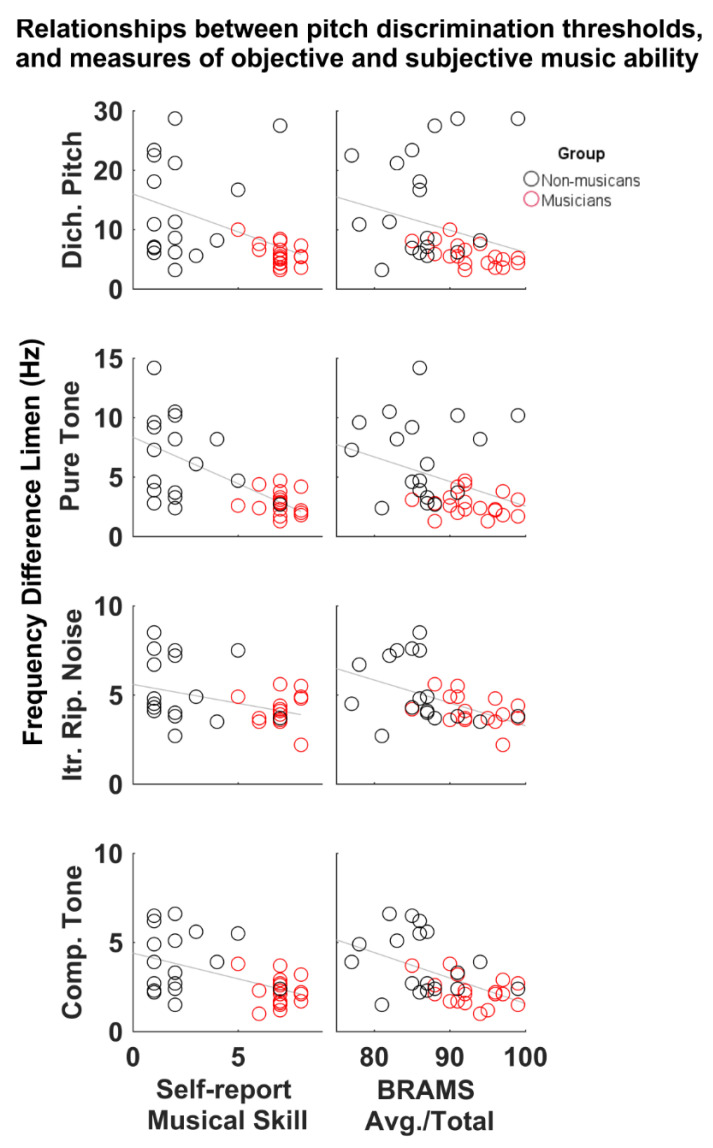
Scatterplots of individual data for musicians (red) and non-musicians (black) with regression lines. Left column shows relationships between pitch discrimination thresholds self-reported (subjective) musical skill (scaled between 1–9, with 1 being novice, 9 professional). Higher self-report is associated with smaller (better) thresholds. Right column shows relationships between pitch discrimination thresholds behavioral scores obtained from the BRAMS musical skills test (objective). Higher score is associated with smaller (better) thresholds.

Pearson’s correlations show that better discrimination thresholds are associated with a higher self-report of musical skill and better scores on all tests of BRAMs musical aptitude. Correlations are reported in Supplementary Table S3. Figure 3 shows individual data for the representative correlations between DLFs, self-report and BRAMS total score. Figure 3 (left column) illustrates that lower (better) DLFs are associated with higher self-reports of musical skill. The spread of the data in Figure 3 also illustrates greater variance in self-report among NM compared to MU, reflecting a wider range of self-assessed musical experience in the NM group. Figure 2 (right column) shows the relationships between DLFs and BRAMS total score. Consistent negative correlations suggest that smaller DLFs are associated with higher musical aptitude.

### 3.3. Discriminant Analysis

A discriminant analysis was conducted to determine which of our variables contributed most to group separation and to test whether an individual’s group category could be correctly identified based on our continuous numeric experimental measures. Continuous variables were DP, PT, IRN and CT DLFs as well as SR Musical Skill and BRAMS avg./total score. Table 2 shows significant mean differences were observed for all variables (*p* < 0.021) except for SR music listening frequency (*p* = 0.774).

The canonical discriminant function showed a significant association between groups and variables; Wilks’ Lambda = 0.145, Chi-square = 60.800, *p* < 0.001, accounting for 85.5% of the between-group variability. Examination of the discriminant loadings (Table 2) showed three significant predictors (i.e., >0.3), namely SR Musical Skill (0.817) and PT DLF (−0.348), and BRAMS avg./total score (0.305). The weakest predictor was IRN DLF (−0.173). Cross-validated classification showed that overall, 89.2% of the subjects were correctly classified into MU and NM groups. It should be noted that log determinants of this analysis showed large differences and Box’s M was significant, suggesting that the assumption of equality of covariance matrices was violated. However, this problem is somewhat allayed given that normality is not a critical assumption for discriminant analysis.

## 4. Discussion

Our results help answer the question of whether perceptual plasticity associated with musical training is specific to certain pitch-related acoustics. Specifically, we have answered two main questions in this study: (1) are musicians better at perceiving specific pitch-related acoustics? and (2) are psychoacoustic thresholds related to objective and subjective measures of music ability?

To answer the first question, an RMANOVA with four within-subject factors of sound type and two between-subject factors of group was conducted using DLF data. Results showed group differences across all sound types, with the greatest differences for dichotic and pure tone stimuli. These data counter our initial hypothesis that temporal encoding mechanisms would be most impacted by musicianship; instead suggesting that music-related plasticity is not restricted to types of pitch-eliciting acoustics. The greatest difference between musician and non-musician discrimination thresholds in the dichotic condition suggests that higher-order mechanisms, such as those requiring a combination of sound across the ears, are greatly impacted by musical training.

Several hypotheses could reasonably explain our findings. One hypothesis is that playing music sharpens one’s ability to extract pitch percepts in conditions where pitch strength is less salient. Whereas the largest threshold difference is in the dichotic condition (less salient pitch), the second largest threshold difference is observed in the pure tone condition, which has the most salient pitch strength. This observation diminishes the saliency hypothesis’ likelihood. A second possibility is that musicians possess a greater aptitude to learn the task than non-musicians, and therefore, perform better overall. A post-hoc examination of the within-session change in threshold showed that non-musicians did have more variability, measured by standard deviation (Supplementary Table S2). However, mean magnitudes of the within-session change in threshold over the four runs did not appear to differ between groups. To verify our observations, we performed two RMANOVAs for stimulus type and group on the within-session change and variability data. Results showed that musicians had lower standard deviation in thresholds to dichotic and pure tone stimuli, compared to non-musicians, but only in the pure and complex tone conditions. No significant differences were observed for within- or between-subject comparisons of the within-session threshold change magnitude. Taken together, these data suggest that acclimatization or learning trajectories from task beginning to end is similar in musicians and non-musicians.

A third possibility is that mechanisms of music-related brain plasticity are not restricted to place or temporal code encoding mechanisms in peripheral or brainstem nuclei [11], but are dominated by central mechanisms [12], or at least beyond the superior olive where dichotic sounds first combine. The current data cannot discriminate between peripheral and central plasticity specifically, however, previous data do suggest a relationship between the two [7,22]. Perhaps music-related plasticity begins in higher-order areas, and gates more peripheral changes, similar to barn owl plasticity [16]. This hypothesis is strengthened by the observation that thresholds are less variable in musicians, suggesting more certainty about the percept.

In answering the second question of whether psychoacoustic thresholds related to objective and subjective measures of music ability, we showed evidence for a relationship between psychoacoustic pitch discrimination and measures of subjective and objective music ability. The correlation data show that discrimination thresholds across all four pitch types were negatively correlated with a higher subjective rating of musicianship, such that individuals who rated themselves with musical ability closer to “professional” on a subjective scale, could hear smaller pitch differences in all four sound conditions. Conversely, individuals who rated themselves with a lower musical ability (i.e., closer to “novice” on the same scale) had greater (poorer) DLFs. To the authors’ knowledge this is the first time that such a relationship has been reported. The results imply that a person’s self-assessment can be a good predictor of their psychoacoustic threshold. It should be noted however, that while the correlations between self-reported music ability and DLF are significant, more than half of the r-values portray a moderately strong relationship (i.e., <0.5); suggesting that other, untested, variables account for additional variance in the relationship. Therefore, while the connection between basic sensory ability and self-assessment of music ability is suggested here, it is only partially accounted for.

Regarding objective measures of music ability, it appears that the online music aptitude tests of melody, pitch, and the average of all music scores showed a consistent relationship with our psychoacoustic test results. In general, higher scores were related to smaller DLFs, suggesting that those who scored well on the BRAMS tests could discriminate sounds with smaller pitch differences. It is interesting to note that although melody and pitch scores correlated with psychoacoustic discrimination thresholds, timing scores did not. Relatedly, melody and pitch were correlated to each other, but neither correlated with timing (see Supplementary Table S3). Timing scores did correlate, however, with SR Musical Skill and BRAMS avg./total score, suggesting that rhythmic ability is related to musical aptitude and self-assessment of musical skill, but may be independent of pitch perception. Taken together, the correlation data show that the ability to discriminate small pitch differences can be reflected in global musical abilities and an individual’s evaluation of their own musical aptitude. This implies that sensory thresholds for pitch discrimination underlie, at least in part, one’s musical ability and self-appraisal of that ability. Furthermore, relationships between sensory threshold for pitch and more broad measures of musicianship are not restricted to a specific mechanism of pitch processing.

The discriminant analysis allowed us to detect the degree to which our variables discriminate between musicians and non-musicians. The variables that contributed most to the predictions of group membership were (1) self-report of musical ability on a scale of 1–9, (2) pure tone DLFs and (3) BRAMS avg./total score. While the relationship between pure tone perception, musical aptitude and musicianship is well established, the contribution of a self-report variable is novel as far as the authors’ knowledge. Here, we show that self-evaluation of musical competence can be meaningfully applied to classify groups and is related to objective measures of music and perceptual ability. Self-evaluation of competence, or self-competence is defined as the sense of one’s capacity [32]. Previous data on this topic show that general self-competence is as associated with measures of cognitive ability such as IQ and academic achievement measured by GPA [33]. Our data support the argument that self-evaluation of competence is a meaningful measure of ability and outcomes [34] and extend into musicianship.

In addition to the finding of self-report as a useful measure, the discriminant analysis showed common characteristics of musicians include psychoacoustic, musical and self-evaluated abilities. This gives rise to the notion that all three areas may interact to define a person who is talented or skilled in music. This is not entirely surprising, given that the benefits of music training vary widely and span from audiometric to multisensory and into cognitive domains across ages [35,36]. It is interesting to note that the self-reported music listening scale did not distinguish between groups. This supports several lines of research showing that active music-making, rather than listening alone, is a catalyst for brain plasticity and internalized perceptual change [22,24,37,38].

In conclusion, this study sheds light on several aspects of plasticity related to musical training. First, we show that the influence of musicianship is not limited to pitch judgements involving monotic/diotic mechanisms but also includes those that rely on dichotic integration. Second, our data show that basic perceptual thresholds are related to measures of both subjective and objective musical ability. Furthermore, and third, the data suggest that self-evaluation of musical ability is a meaningful part of musicianship such that high evaluation of competence are characteristic of musician group members. Taken together, the data update the neurobehavioral profile of musicians and extend creative ability measurements into new arenas.

## Figures and Tables

**Table 1:** Group characteristics of music education, experience and skill in non-musicians and musicians.

		Self-Reported Music Education (yrs.)			Self-Reported Musical Skill and Listening (Scale 1–9)		Objective Musical Skill Scores (MBEA) (%)			
Group	Metric	Age at Test	Age Began Music	Total Music Education	SR Musical Skill	SR Music Listening Frequency	Melody	Timing	Pitch	Total/Avg.
Non-musicians (n = 18)	Mean	25.11	7.63	5.13	2.22	7.00	84.28	86.89	88.06	86.22
	Std. Dev.	1.906	2.875	2.416	1.629	1.680	7.466	6.296	11.254	5.342
Musicians (n = 20)	Mean	23.75	7.60	12.20	7.05	7.20	90.90	93.10	95.75	92.9
	Std. Dev.	5.543	3.202	4.099	0.759	1.609	6.782	4.909	4.541	3.782

Self-reported music education, music skill and listening frequency measures obtained via questionnaire and are reported in years. Only 8 non-musicians had previous music education. Self-reported music skill was rated on a scale from 1–9, with 1 being “novice” and 9 denoting “professional”. Music listening frequency was rated on a scale from 1–9 with 1 being “never” and 9 “all the time”. Melody, timing, pitch and average/total musical skill scores obtained via online aptitude test (www.brams.org (accessed on 13 September 2018)) and are reported in percent correct.

**Table 2:** Discriminant analysis results including tests of equality of group means and variable loadings.

Metric	Wilks’ Lambda	F	df1	df2	Sig.	Structure Matrix (Loadings)
Dich. Pitch DLF	18.502	18.502	1	36	<0.001	−0.295
Pure Tone DLF	25.668	25.668	1	36	<0.001	−0.348 *
Itr. Rip. Noise DLF	6.333	6.333	1	36	0.016	−0.173
Comp. Tone DLF	16.646	16.646	1	36	<0.001	−0.280
SR Musical Skill	141.793	141.793	1	36	<0.001	0.817 *
BRAMS Avg./Total	19.747	19.747	1	36	<0.001	0.305 *

Structure matrix (loadings) shows pooled within-groups correlations between discriminating variables and standardized canonical discriminant functions, * denotes important correlations >0.3.

## Supplementary Materials

The following are available online at https://www.mdpi.com/article/10.3390/brainsci11121592/s1, Table S1: Shapiro-Wilk Tests of normality for threshold measures by stimulus type, Table S2: Mean threshold and within-session change measures by stimulus type in Non-musicians and Musicians, Table S3: Correlations between frequency discrimination limen thresholds and behavioral measures (n = 38).
