# Supplementary material for: Diotic and Dichotic Mechanisms of Discrimination Threshold in Musicians and Non-Musicians
Source: Brain Sci. 2021 Nov 30;11(12):1592. doi: 10.3390/brainsci11121592 (PMC8699398; doi:10.3390/brainsci11121592)
Supplement: Supplementary file 1 [file brainsci-11-01592-s001.zip › brainsci-1446377-supplementary.pdf]

brain sciences

MDPI

Supplementary Table S1.

**Supplementary Table S1. Shapiro-Wilk Tests of normality for threshold measures by stimulus type**				
**Metric**	**Group**	**Statistic**	**df**	**Sig.**
Dich. Pitch DLF	Non-musicians	.883	18	.029
	Musicians	.952	20	.487
Pure Tone DLF	Non-musicians	.924	18	.155
	Musicians	.952	20	.395
Itr. Rip. Noise DLF	Non-musicians	.861	18	.013
	Musicians	.902	20	.045
Comp. Tone DLF	Non-musicians	.901	18	.059
	Musicians	.957	20	.487
SR Musical Skill	Non-musicians	.737	18	**.000**
	Musicians	.796	20	**.001**
BRAMS Avg./Total	Non-musicians	.959	18	.583
	Musicians	.970	20	.758

Supplementary Table S2.

Supplementary Table S2. Mean threshold and within-session change measures by stimulus type in Non-musicians and Musicians

		Mean discriminant frequency threshold over four consecutive runs				Within-session variation in threshold				Within-session threshold shift from first to last run			
Group	Metric	Dich. Pitch DLF	Pure Tone DLF	Itr. Rip. Noise DLF	Comp. Tone DLF	Dich. Pitch SD	Pure Tone SD	Itr. Rip. SD	Comp. SD	Dich. Pitch ΔDLF	Pure Tone ΔDLF	Itr. Rip. ΔDLF	Comp. ΔDLF
Non-musicians (n=18)	Mean	14.49	6.76	5.16	3.90	4.72	2.98	1.43	1.02	.35	-1.05	.34	-.19
	Std. Dev.	8.86	3.44	1.81	1.69	3.80	2.30	2.07	.75	6.67	5.66	2.57	1.26
Musicians (n=20)	Mean	5.79	2.70	4.04	2.19	2.15	1.12	.92	.65	1.29	-.04	-.16	.06
	Std. Dev.	1.79	.99	.80	.79	1.27	1.33	1.08	.41	3.50	1.87	1.18	.85

Supplementary Table S3.

Supplementary Table S3. Correlations between frequency discrimination limen thresholds and behavioral measures (n=38)

Metric	Group	Dich. Pitch DLF	Pure Tone DLF	IRN DLF	Comp. Tone DLF	Self-Report Musical Skill	Self-Report Music Listening	Melody	Timing	Pitch	Total
Dich. Pitch DLF	Pearson Corr.	1	**0.65**	**0.41**	**0.43**	**-0.46**	-0.04	-0.22	-0.13	-0.26	**-0.29**
	Sig.		**0.00**	**0.01**	**0.01**	**0.00**	0.80	0.18	0.43	0.12	**0.08**
Pure Tone DLF	Pearson Corr.		1	**0.63**	**0.73**	**-0.67**	-0.14	**-0.31**	-0.13	**-0.36**	**-0.38**
	Sig.			**0.00**	**0.00**	**0.00**	0.41	**0.06**	0.44	**0.03**	**0.02**
Itr. Rip. Noise DLF	Pearson Corr.			1	**0.83**	**-0.40**	-0.14	**-0.49**	**-0.32**	**-0.28**	**-0.50**
	Sig.				**0.00**	**0.01**	0.41	**0.00**	**0.05**	**0.09**	**0.00**
Comp. Tone DLF	Pearson Corr.				1	**-0.54**	-0.04	**-0.46**	**-0.36**	**-0.37**	**-0.54**
	Sig.					**0.00**	0.81	**0.00**	**0.03**	**0.02**	**0.00**
Self-Report Musical Skill	Pearson Corr.					1	0.07	**0.40**	**0.45**	**0.55**	**0.63**
	Sig.						0.69	**0.01**	**0.01**	**0.00**	**0.00**
Self-Report Music Listening	Pearson Corr.						1	0.07	0.10	0.17	0.15
	Sig.							0.67	0.55	0.30	0.36
Melody	Pearson Corr.							1	0.19	**0.42**	**0.81**
	Sig.								0.25	**0.01**	**0.00**
Timing	Pearson Corr.								1	0.17	**0.52**
	Sig.									0.31	**0.00**
Pitch	Pearson Corr.									1	**0.78**
	Sig.										**0.00**
